# Lack of neural contributions to the summating potential in humans with Meniere’s disease

**DOI:** 10.3389/fnins.2022.1039986

**Published:** 2022-12-07

**Authors:** William J. Riggs, Tatyana E. Fontenot, Meghan M. Hiss, Varun Varadarajan, Aaron C. Moberly, Oliver F. Adunka, Douglas C. Fitzpatrick

**Affiliations:** ^1^Department of Otolaryngology-Head and Neck Surgery, The Ohio State University, Columbus, OH, United States; ^2^Department of Audiology, Nationwide Children’s Hospital, Columbus, OH, United States; ^3^Department of Otolaryngology-Head and Neck Surgery, University of North Carolina at Chapel Hill, Chapel Hill, NC, United States

**Keywords:** electrocochleography, summating potential, Meniere’s disease, auditory nerve, inner hair cells, outer hair cells

## Abstract

**Objective:**

To investigate the electrophysiology of the cochlear summating potential (SP) in patients with Meniere’s disease (MD). Although long considered a purely hair cell potential, recent studies show a neural contribution to the SP. Patients with MD have an enhanced SP compared to those without the disease. Consequently, this study was to determine if the enhancement of the SP was in whole or part due to neural dysfunction.

**Design:**

Study participants included 41 adults with MD and 53 subjects with auditory neuropathy spectrum disorder (ANSD), undergoing surgery where the round window was accessible. ANSD is a condition with known neural dysfunction, and thus represents a control group for the study. The ANSD subjects and 17 of the MD subjects were undergoing cochlear implantation (CI) surgery; the remaining MD subjects were undergoing either endolymphatic sac decompression or labyrinthectomy to alleviate the symptoms of MD. Electrocochleography was recorded from the round window using high intensity (90 dB nHL) tone bursts. The SP and compound action potential (CAP) were measured to high frequencies (> = 2 kHz) and the SP, cochlear microphonic (CM) and auditory nerve neurophonic (ANN) to low frequencies. Linear mixed models were used to assess differences between MD and ANSD subjects.

**Results:**

Across frequencies, the MD subjects had smaller alternating current (AC) response than the ANSD subjects (*F* = 31.6_1_,_534_, *p* < 0.001), but the SP magnitudes were larger (*F* = 94.3_1_,_534_, *p* < 0.001). For frequencies less than 4 kHz the SP magnitude in the MD group was significantly correlated with the magnitude of the CM (*p*’s < 0.001) but not in the ANSD group (*p*’s > 0.05). Finally, the relative proportions of both ANN and CAP were greater in MD compared to ANSD subjects. The shapes of the waveforms in the MD subjects showed the presence of multiple components contributing to the SP, including outer and inner hair cells and neural activity.

**Conclusion:**

The results support the view that the increased negative polarity SP in MD subjects is due to a change in the operating point of hair cells rather than a loss of neural contribution. The steady-state SP to tones in human subjects is a mixture of different sources with different polarities.

## Introduction

Meniere’s Disease (MD) is a disorder of the inner ear characterized by audio-vestibular symptoms including recurrent episodes of dizziness, aural fullness, and tinnitus followed by fluctuating degree of sensorineural hearing loss (SNHL) (Basura et al., 2020). Temporal bones from subjects diagnosed with MD typically have distention of endolymphatic spaces including the cochlear duct, consistent with endolymphatic hydrops (EH), which then causes compression of the tectorial membrane and organ of Corti (Hallpike and Cairns, 1938; Paparella, 1984; Rauch et al., 1989; Salvinelli et al., 1999). It is thought that this compression is responsible for the primary electrophysiological change associated with MD, which is an increased summating potential (SP) in electrocochleography (ECochG) signals. To tones, the SP is a baseline shift, or direct current (DC) potential, during acoustic stimulation. The idea is that the compression of the tectorial membrane with EH causes the operating point of hair cells to become more asymmetric, or more likely to saturate for movement in one direction relative to the other. Asymmetric saturation produces even-order harmonics distortion, and DC, or zero frequency, is an even-order distortion, resulting in the SP being larger for a given input (Teich et al., 1989).

Various ECochG techniques have been used to measure the increased SP, with recording sites of the active electrode including the skin of the external auditory canal (Coats and Dickey, 1970; Coats, 1981a), the tympanic membrane (Stypulkowski and Staller, 1987; Ruth et al., 1988), and the cochlear promontory *via* transtympanic needle placement (Schmidt et al., 1974; Gibson et al., 1983). In addition to the SP, ECochG signal components include the cochlear microphonic (CM), an alternating current (AC) potential produced by hair cells in response to motion of the basilar membrane, the compound action potential (CAP), produced by synchronous firing of auditory nerve fibers to the onsets of sounds, and the auditory nerve neurophonic (ANN), an AC potential to low frequencies from phase-locked firing of auditory nerve fibers. In response to a click, the SP is seen as a shoulder prior to the CAP (Coats, 1981b; Durrant and Ferraro, 1991). However, the amplitude and polarity of the SP can vary depending on recording site, stimulus frequency and intensity (Dallos et al., 1972; Ferraro and Durrant, 2006) as well as changes in the hair cell transducer operating point (Kirk and Patuzzi, 1997). Animal studies have shown the SP to be comprised of both outer and inner hair cell sources (OHCs and IHCs, respectively), with IHCs having a more asymmetric operating point than the OHCs, thus producing a larger contribution to SP per hair cell than OHCs (Dallos et al., 1972; Zheng et al., 1997; Durrant et al., 1998).

The motivation for the current study stems from recent findings of changes in the SP in gerbils following kainic acid application to the round window (RW) (Pappa et al., 2019). This result supported previous indications that the SP is not a purely hair cell potential, instead the auditory nerve also contributes (van Emst et al., 1995; Sellick et al., 2003; Forgues et al., 2014). Specifically, the SP to tone bursts typically became more negative after application of a neurotoxin, indicating that a neural contribution with a positive polarity was removed. Recently, further studies with multiple neurotoxins have shown that the neural component can be subdivided into a “dendritic” contribution produced from excitatory postsynaptic potentials in auditory nerve dendrites, and a “spiking” component produced by the action potentials (Lutz et al., 2022). Because the net neural potential has positive polarity, the increase in negative polarity to tone bursts seen in MD subjects could be due to one of two possible mechanisms, either increased asymmetry in the operating point of HC transduction caused by the EH (Gibson, 2009; Iseli and Gibson, 2010), or to loss of the positive neural contribution associated with hearing loss. Recently, loss of hearing deduced through loss of neural potentials, has been associated with endolymphatic hydrops induced in guinea pigs (Lee et al., 2020). Thus, for this study we sought to address the mechanism of SP production in Meniere’s subjects by comparing the results to subjects with known neural dysfunction. This control population has been diagnosed with auditory neuropathy spectrum disorder (ANSD), a condition with hearing loss secondary to neural dysfunction while the cochlear function remains intact. We have previously shown that the SP in ANSD subjects is large and generally negative compared to pediatric and adult cochlear implant subjects receiving cochlear implants, and that the increased SP in ANSD subjects is correlated with a lack of neural contributions (Riggs et al., 2017). The rationale to discriminate among the proposed mechanisms in MD subjects was to test if the size of the SP in MD subjects was comparable to that of the ANSD subjects, and neural contribution in the form of a CAP or ANN were similarly lacking. If so, then the increased negative SP in MD subjects could be in part due to the lack of a neural contribution. In contrast, if the enhancement of the SP was greater in MD than in ANSD subjects and the neural contribution was not similarly reduced, then the increased asymmetry in the hair cell transducers is the more likely mechanism for the increased SP in MD subjects.

## Materials and methods

### Participants

This study was approved by the local biomedical Institutional Review Boards at the Ohio State University and University of North Carolina at Chapel Hill. All participants provided written consent and assent (when applicable) after being informed of the purpose of the study, procedures and potential risks. Study participants included 41 adults with MD and 53 (50 pediatric and 3 adults) subjects diagnosed with auditory neuropathy spectrum disorder (ANSD), undergoing surgical treatment where the round window was accessible. This dataset is the same as that reported in Riggs et al. (2017) with the addition of 5 pediatric subjects. All participants in the MD group had medical record documentation of meeting the American Academy of Otolaryngology Head and Neck Surgery’s 2020 position guidelines for classifications of definite MD, these were: (a) two or more definitive spontaneous episodes of vertigo lasting at least 20 min, (b) documented hearing loss determined by audiometry on at least one occasion, (c) tinnitus, or (d) aural fullness in the ear of concern (Basura et al., 2020). Subjects in the ANSD group had clinical documentation of ANSD as defined by abnormal Wave V and presence of CM in auditory brainstem response (ABR), indicating decreased nerve function but intact hair cell function. ECochG was performed at the time participants were undergoing surgical management (CI surgery, endolymphatic sac decompression/shunt, or labyrinthectomy for MD subjects; cochlear implantation for ANSD subjects).

### Surgery and electrophysiology

Electrocochleography recordings were obtained for all patients intraoperatively at the time of surgical intervention. A cortical mastoidectomy was performed followed by a transmastoid facial recess approach for all procedures (CI, endolymphatic sac decompression/shunt, labyrinthectomy). Prior to endolymphatic sac opening, labyrinthectomy, or to round window opening/electrode insertion (for CI surgery), a monopolar probe (Kartush raspatory probe, Plainsboro, NJ, USA, or Neurosign 3602-00-TE, Magstim Co., Wales, UK) was positioned at the RW niche. The evoked signal was recorded differentially with a common surface electrode (Neuroline 720, Ambu Inc., Ballerup, Denmark) placed on the forehead and a reference electrode placed at the contralateral mastoid. Stimulation and recording of evoked responses were controlled using a Bio-logic Navigator Pro (Natus Medical Inc., San Carlos, CA, USA) evoked potential system. Stimuli were delivered through an insert transducer (ER3b, Etymotic Inc., Elk Grove Village, Illinois, USA) connected through a sound tube to a foam insert earphone placed in the external auditory canal.

The stimulation approach for each patient was to administer a series of alternating (rarefaction and condensation starting phase) tone bursts of 0.25, 0.5, 0.75, 1, 2, 4 kHz at 90 dB nHL (101–115 dB peak SPL depending on frequency) at a rate of 17.3 Hz (0.25–1.0 kHz) and 23.3 Hz (2–4 kHz) for 500 averages (250 per phase). Stimulation levels were calibrated in units of dB peak equivalent sound pressure level (peSPL) using a 1 inch 2 cc coupler routed to a sound level meter (System 824, Larson Davis, Depew, NY, USA) set to fast mode. A Blackman window was used and rise and fall times consisted of one cycle for frequencies of 0.25–1 kHz and 1 ms for 2 and 4 kHz tone bursts. Plateaus were 5–20 cycles (lower frequencies had fewer cycles than higher frequencies). The filter setting for the high-pass was set at 10 Hz and for the low-pass was at 5 kHz (0.25–1 kHz tone bursts), 10 kHz (2 kHz tone burst), or 15 kHz (4 kHz tone burst). Signals were amplified at 50,000× with artifact rejection level set at 47 μV, or if necessary raised due to large magnitude of ECochG signals (typically in ANSD participants).

### Data analysis

Electrocochleography results were analyzed using MATLAB R2021a (Mathworks Corp., Natick, MA, USA) with custom software procedures. The condensation and rarefaction traces were used to calculate the DIF and SUM waveform, which were the difference of the two phases or their sum, each divided by 2. The DIF waveform contains odd harmonics of the fundamental frequency, or those that alternate with the phase of the stimulus, while the SUM contains the even harmonics, or those that do not change with stimulus phase. The mixture of hair cell and neural components in the SUM and DIF is complex. The response from hair cells, both OHCs and IHCs, is the CM which alternates with the stimulus phase, and thus the largest component of the CM appears in the DIF. However, if the operating point, or position on the transducer input/output function when at rest, is displaced from halfway, then asymmetric saturation occurs to the two phases of stimulation and even harmonics of the CM appear in the SUM. Typically, the operating point of OHCs is considered slightly asymmetric but close to the 50% point, while inner hair cells are more asymmetric (Russell, 2008; Johnson, 2015). Evidence to support greater asymmetry in the IHC contribution to the CM is seen in animals using ototoxins to remove OHCs (Pappa et al., 2019). The neural potential to frequencies within the phase-locking range (<∼1500 Hz) is the ANN, which is also periodic to the stimulus so a large proportion of the ANN appear in the DIF (Fontenot et al., 2017). However, because the spike rate cannot go below zero, the distribution of action potentials that produce the ANN is rectified, which is an asymmetric saturation that produces a larger proportion to appear in the SUM than is the case for the CM. It is the degree of asymmetries in both hair cell and neural potentials that produces the SP, or shift from baseline in the time waveform, once the response reaches steady state. A second neural potential is the CAP, produced by well-timed action potentials to the onset of sounds. To the degree the shape of the CAP does not change with phase it is primarily an asymmetric response that appears in the SUM. However, to low frequencies the rising phase of the stimulus is shifted enough along the time axis that a proportion of the CAP appears in the DIF. In addition, the slow risetime (one period to frequencies 1 kHz and below) results in less synchrony to the stimulus onset and a smaller CAP to low compared to high frequencies.

**FIGURE 1:**
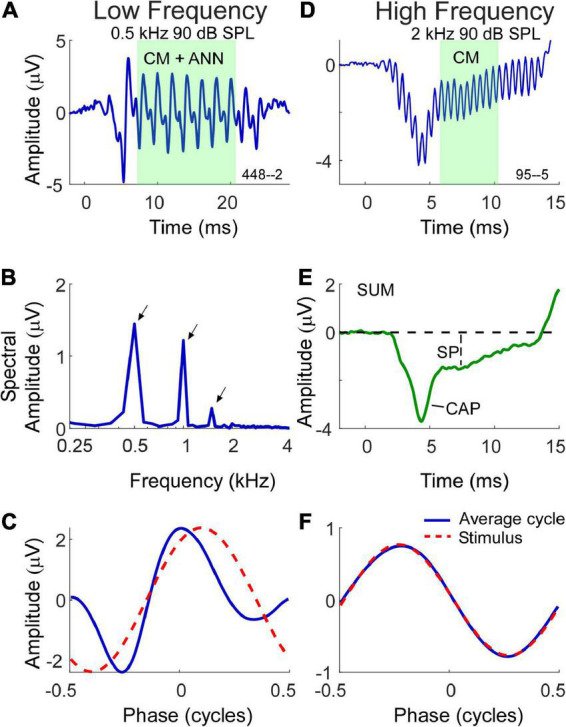
Morphology of electrocochleography (ECochG) signal waveforms in response to low and high frequency stimuli. **(A)** Waveform of the ECochG response to a 500 Hz stimulus at 90 dB SPL. The ongoing portion used for analysis is highlighted, and at this frequency consists of both the cochlear microphonic (CM) and auditory nerve neurophonic (ANN) (when present). **(B)** The fast fourier transform of the ongoing portion of the waveform in panel **(A)** with first three harmonic peaks (500 Hz, 1 kHz, 1.5 kHz) of statistical significance indicated by arrows. **(C)** The shape of the response in the form of an average cycle (blue line), which is the average of all cycles in the ongoing portion of the ECochG response. The red dashed waveform represents the sinusoidal morphology of the stimulus shifted to the same phase as the response, as determined from a cross correlation of the two. **(D)** Waveform of the ECochG response to a 2 kHz stimulus at 90 dB SPL. At this frequency, the ongoing portion of response is composed of the CM only. **(E)** The SUM response is the average response to the two phases of stimulation. It reduces the CM and highlights the compound action potential (CAP) and summating potential (SP). **(F)** Unlike the average cycle to low frequencies which contains an ANN, the average cycle to high frequencies is relatively undistorted.

With these factors in mind, the methods of data collection used in this study are shown in Figure 1. Two examples are shown, one a response to a low frequency tone (Figures 1A–C) and one to a high frequency tone (Figures 1D–F). For clarity, the time waveforms (Figures 1A,D) are to condensation phase only, which was alternately presented with rarefaction phase stimuli. The AC response was measured during the time windows shown (green) which were chosen to be an integer number of cycles after the CAP and prior to stimulus offset. To the low frequency stimulus this AC response is a mixture of the CM and ANN (when present), while the response to the high frequency is to the CM only. The measure of AC magnitude we used is shown in the spectrum (Figure 1B), obtained using Fourier methods. Here, three peaks shown here for condensation phase are present corresponding to amplitudes of the 1st, 2nd, and 3rd harmonics. To make use of all the stimuli the peaks of the 1st and 3rd harmonics were measured from the DIF, and the 2nd from the SUM, for all cases. Each peak was considered significant if (1) it was more than three standard deviations above the noise floor, as measured from six bins, three on each side of the peak of interest, and (2) if the peaks to each cycle when plotted as a cycle histogram were significant at *p* < 0.01 based on the Rayleigh criterion (Mardia and Jupp, 1999; Berens, 2009). Responses that were not significant were noted but not included in calculations.

In most of the data presented we will use the combined CM and ANN to low frequencies, but to assess the neural contribution we need to separate the CM and ANN. Detailed description of our approach to this difficult problem has been previously published (Fontenot et al., 2017) and is based on the shapes of the cyclic response expected from the different biophysical properties that produce each. Briefly, the input/output function of the CM can be viewed as a second-order Boltzmann function with a variable operating point that allows for asymmetric saturation to the two directions of stereociliary motion. Like the CAP, the ANN can be modeled as the convolution of a unit potential, or average shape of an action potential as it appears at the round window, with the distribution of all action potentials as seen in a population post-stimulus time histogram (PSTH). To low frequencies this PSTH is cyclic and rectified due to phase locking, so the cycle histogram is an equivalent version. We introduce a parameter “spread-of-excitation” (SOE), which affects the width of the rectification in order to capture any increased spread across the population, thus representing variance in extent of the cochlea responding. To visualize the cyclic response, each cycle in the ongoing response (box in Figure 1A) is used to produce the “average cycle” (Figures 1C,F). Again, these are to the condensation phase only, but in practice the rarefaction response can be shifted to use all the cycles. For the low frequency response (Figure 1C) the average cycle shows distortions that cannot be well fit by a model of the CM but can be well fit by the combination of CM and ANN, and the proportion of each required to produce the best fit operates as an estimate for the magnitude of each in the original signal. In this case the model returned an ANN that was 42% of the AC response. To the high frequency stimulus (Figure 1F) the average cycle is sinusoidal, and no ANN is present. The relationship between the CM and ANN in the average cycle was demonstrated in animal studies where the neural elements could be removed pharmacologically (Fontenot et al., 2017).

The SP magnitude and its polarity in response to the tone bursts was measured from the SUM waveforms (Figure 1E) as the sustained deflection from baseline to a point after steady state was reached (Ferraro and Durrant, 2006; Gibson, 2009). The 10 Hz high-pass filter can cause a decline toward zero as a function of duration, so the measurement window was as soon after the CAP (if present) that could be considered steady state. The CAP was also typically most prominent in the SUM (Figure 1E), however, as mentioned to low frequencies a proportion could also appear in the DIF.

In previous reports on CI subjects, including the ANSD subjects included here, the presence or absence of a CAP was noted visually but for several reasons was difficult to quantify (Campbell et al., 2015; Scott et al., 2016; Fontenot et al., 2022). These reasons include (1) the lack of a CAP in approximately 50% of CI subjects, (2) the presence of primarily low frequency responses where the CAP is often small, can be partially present in the DIF rather than the SUM curve (as described above), and is difficult to filter for because the frequency range of the CM overlaps that of the CAP, and (3) it can ride on a rising or falling SP making measurement difficult. In general, the presence of a CAP is more convincing at high frequencies than low, so our visual determinations are reported for high frequency (2 and 4 kHz) only.

### Statistics

Data were analyzed using MATLAB and SPSS 24 (IBM Corp., Armonk, NY, USA). Linear mixed models (LMMs) were used to assess differences across MD and ANSD subjects, with subject as a random factor and frequency and group (MD or ANSD) as fixed factors. Outcome measures included the AC response magnitude (CM + ANN for frequencies < 2 kHz and CM only for higher frequencies), the SP, the proportion of ANN vs. CM and presence or absence of a CAP. *Post-hoc t*-tests were two-tailed with statistical significance determined at the 95% confidence level and corrected for multiple comparisons.

## Results

This study included 41 adult subjects with MD (ages 37–71, mean 57.7 ± 12.89 years standard deviation) and 53 ANSD subjects, 50 who were children (1–11, mean 3.6 ± 2.77 years) and three adults (22, 53, and 58 years).

### Audiometric thresholds

**FIGURE 2:**
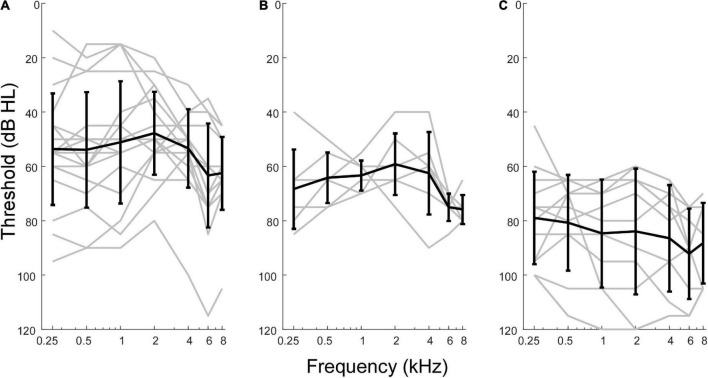
Audiometric profiles of subjects with Meniere’s disease separated by type of surgical intervention. **(A)** Subjects undergoing hearing preservation procedures of endolymphatic sac decompression and shunt placement had thresholds closest to normal levels and greatest variability in audiometric thresholds. **(B)** Subjects undergoing labyrinthectomy had hearing loss between the other two operative groups. **(C)** Subjects undergoing cochlear implant procedures had thresholds indicative of most severe hearing loss overall.

Although pooled in later analyses, there were differences in hearing between the three groups of MD subjects included (Figure 2). Those undergoing ELS surgery, which does not compromise hearing, had the widest range of pre-operative thresholds, encompassing normal thresholds at some frequencies to profound hearing loss in other cases. Subjects undergoing labyrinthectomy had a relatively narrow range of thresholds showing mostly moderate levels of hearing loss. The CI subjects had more severe hearing loss overall. In terms of pure tone average (PTA, five frequencies, 0.25, 0.5, 1, 2, and 4 kHz) the means for ELS and labyrinthectomy were similar (51.6 and 62.2 dB HL), but the standard deviations differed (18.92 and 5.65 dB), while the mean PTA for the CI subjects was poorer (86.0 ± 19.52 dB HL). The ANSD subjects were all receiving CIs, and the hearing was similar to that of the CI group of Meniere’s subjects (mean PTA of 88.0 ± 19.03 dB HL).

### Distributions of alternating current responses and summating potentials across cases

**FIGURE 3:**
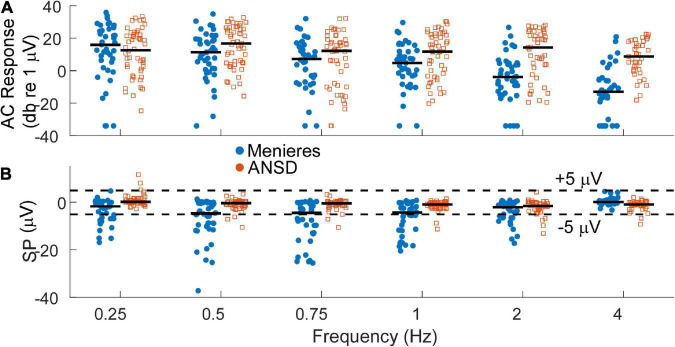
**(A)** Distribution of alternating current (AC) response magnitudes by frequency for the Meniere’s disease (MD) (filled blue circles) and auditory neuropathy spectrum disorder (ANSD) (open orange squares) groups. The AC response is measured as the sum of peaks in the spectrum to the first 3 harmonics, if significant compared to surrounding frequencies. To low frequencies in the phase-locking range it can consist of the cochlear microphonic (CM) and auditory nerve neurophonic (ANN) if there is an ANN present, while to high frequencies it consists of the CM. Black bars indicate the group mean. **(B)** Distribution of the summating potential (SP) magnitude by frequency for both groups. Black bar indicates the group means. Dashed lines indicate ±5 μV, roughly separating small from large SPs.

Auditory neuropathy spectrum disorder subjects comprise a human model of neural dysfunction with an assumed near normal population of OHCs. In CI subjects they have among the largest total response, or sum of responses across frequency, particularly higher frequencies (> = 2 kHz), indicating abundant hair cell responses compared to other CI recipients (Riggs et al., 2017). They thus form a reasonable group for comparison with MD subjects to test the hypothesis that loss of neural function is responsible for the large SP reported in the literature for MD subjects. As shown in Figure 3A, the largest AC responses in ANSD subjects receiving CIs were comparable to the largest with MD, even though many of the MD subjects had minimal hearing loss (Figure 2A). Greater loss of cochlear function is visible across all frequencies except 250 Hz in Figure 3A.

At least in part because of a lack of neural contribution, the SP of many ANSD subjects to high frequencies is generally larger in magnitude than the SP in subjects with other causes of SNHL, and these large SPs are consistently negative in polarity (Riggs et al., 2017). In Figure 3B, the SP of many Meniere’s and most ANSD subjects were (< ± 5 μV, dashed lines). However, some of the SPs in both groups were larger than 5 μV and most of those had a negative polarity. The largest SP magnitudes were consistently seen in the MD subjects and primarily to low frequencies.

The AC and SP distributions were assessed with LMMs that had fixed factors of frequency and group (MD or ANSD) and subject as a random factor. For the AC response, the model showed significant effects of frequency (*F* = 11.37_5_,_534_, *p* < 0.001) and group (*F* = 31.25_1_,_534_, *p* < 0.001), as did the LMM for the SP (frequency, *F* = 10.00_5_,_534_, *p* < 0.0.001, SP, *F* = 94.34_1_,_534_, *p* < 0.001). There was also a significant interaction between group and frequency for both the AC responses (*F* = 6.41_5_,_534_, *p* < 0.001) and SPs (*F* = 10.78_5_,_534_, *p* < 0.001). We note here that age is a confounding factor in this analysis. Since the ages of the two groups is nearly entirely separate (3 adult ANSD subjects overlap in age with the MD subjects) the statistics using age in the LMMs rather than group are nearly identical.

### Relationships between alternating current responses and summating potentials

**FIGURE 4:**
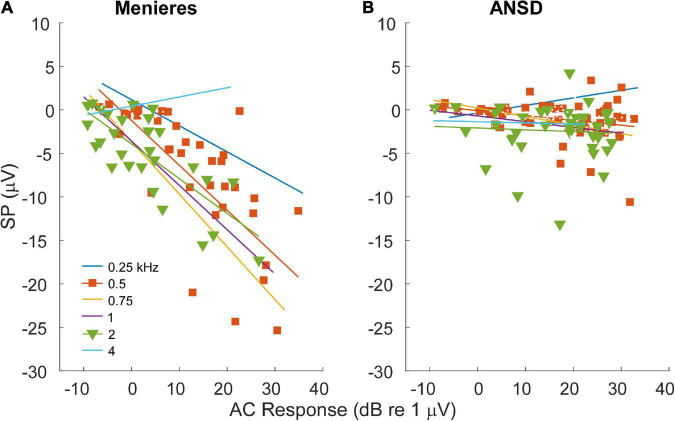
Relationship between alternating current (AC) response and summating potential (SP) magnitudes in electrocochleography (ECochG) responses across all tested frequencies. **(A)** ECochG signals of subjects with Meniere’s disease (MD) demonstrated strong correlation between the magnitude of the AC response and the magnitude (negative polarity) of the SP in all frequencies except 4 kHz (see Table 1). **(B)** In subjects with auditory neuropathy spectrum disorder (ANSD), an increased size of the AC response showed much smaller trends, although in the same direction of an increase in a negative SP for all frequencies other than 4 kHz (see Table 1).

For MD subjects, there was a relationship between the size of the AC response and magnitude of the SP (Figure 4A). For clarity the data are shown for only two frequencies, while the regression lines are shown for each. The data for the regressions are shown in Table 1. There was an increasingly negative SP as the AC response increased for all frequencies except 4 kHz, with the correlation (r) to the 750 Hz stimulus. In contrast, in ANSD subjects the size of the SP was not strongly correlated to the size of the AC response for any frequencies, and had slopes close to zero that could be either negative or positive. The difference between MD and ANSD subjects suggests that the negative SPs in MD are not the result of loss of neural contributions, or the results would be expected to be similar to ANSD cases.

**TABLE 1:** Relationships between the summating potential (SP) and alternating current (AC) response magnitude.

Freq (kHz)	Meniere’s			ANSD		
	*n* (%)^1^	*r*	*p*	*n* (%)^1^	*r*	*p*
0.25	36 (87.8)	0.649	< 0.001	46 (86.8)	0.404	0.004
0.5	38 (92.7)	0.615	< 0.001	51 (96.2)	0.285	0.041
0.75	33 (80.5)	0.768	< 0.001	36 (67.9)	0.392	0.015
1	35 (85.4)	0.739	< 0.001	45 (84.9)	0.371	0.011
2	28 (68.3)	0.765	< 0.001	38 (71.7)	0.065	0.697
4	15 (36.6)	0.537	0.039	37 (69.8)	0.057	0.736

^1^Number and percent of cases that had an AC response > −10 dB (re 1 uV) to each frequency. Smaller responses were omitted from the correlations because the SP was always near zero.

### Neural responses in Meniere’s disease and auditory neuropathy spectrum disorder subjects

**FIGURE 5:**
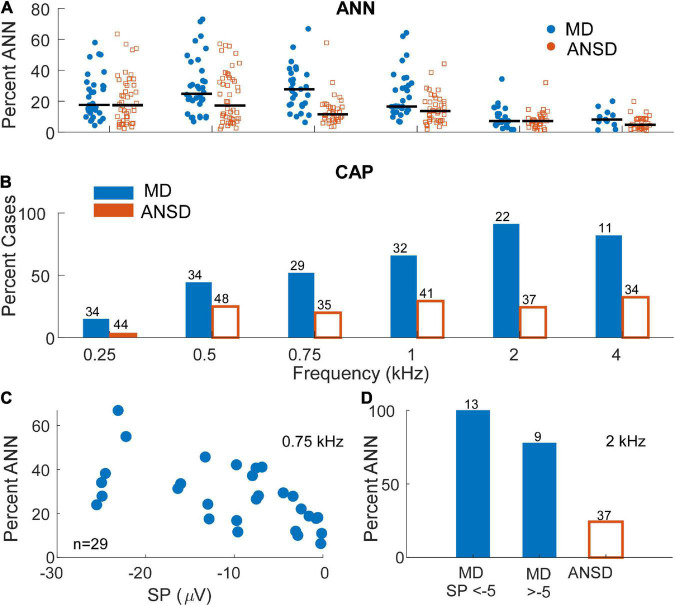
Distribution and prevalence of markers of neural activity in electrocochleography (ECochG) responses of the tested populations. **(A)** The percentage of the alternating current (AC) response attributable to auditory nerve neurophonic (ANN) in subjects with auditory neuropathy spectrum disorder (ANSD) and Meniere’s disease (MD) across frequencies. As expected, signals in response to stimuli between 250 and 1000 Hz demonstrated the greatest evidence of significant ANN contribution in both populations with signals recorded in Meniere’s subjects demonstrating relatively higher range of neural activity at 500 and 750 Hz. Black bars represent mean percentage of ANN contribution in each group. **(B)** The proportion of the ECochG signals in both tested populations that demonstrated a compound action potential (CAP). Signals recorded in subjects with MD demonstrated an identifiable CAP in most subject, in contrast to ANSD subjects where the proportion was small. The proportion of cases with a CAP was greater for higher frequencies in the subjects with MD. **(C)** The proportion of the ANN for MD cases as a function of the summating potential (SP) for one frequency (750 Hz) where the ANN was relatively strong [as in panel **(A)**] and there were many cases with large SPs (as in Figure 3B). **(D)** The proportion of the CAP separated by SPs above and below –5 μV in response to a high frequency tone burst (2 kHz). In both panels **(A,B)** the neural activity was high in cases with both large and small SPs.

To further test the hypothesis that a loss of neural responses could be responsible for the large, negative SP in MD subjects, data showing the comparison of neural responses in the two groups is shown in Figure 5. In Figure 5A, the percentage of the AC response due to the ANN as estimated by our modeling approach (see Section “Materials and methods”) is shown. In general, the proportion of the responses consisting of the ANN was higher in MD than in ANSD subjects, and a LMM showed the increase as a function of group (MD or ANSD) to be significant (*F* = 17.7_1_,_395_, *p* < 0.001), as was the effect of frequency (*F* = 18.6_5_,_395_, *p* < 0.001). The lower number of data points compared to Figure 4 is because only AC responses >0.5 μV (−6 dB re 1 μV) were included. Smaller responses are too noisy for the model to fit appropriately. Similarly, in Figure 5B, data on the presence or absence of the CAP shows that it was commonly present in MD subjects and relatively rare in ANSD subjects. This data must be approached with some caution because the CAP, while often obvious, can sometimes be difficult to measure or even to discern (Scott et al., 2016). In addition, to low frequencies, as described in the Section “Materials and methods,” its largest component can appear in the DIF rather than the SUM curve. Finally, as will be further discussed below, there is often a “CAP-like” shape in the SUM response that is due to convergence of different sources with different timing and polarities (Pappa et al., 2019; Lutz et al., 2022). Despite the difficulties, the differences regarding the CAP are so large that it is clear MD subjects are much more likely to show neural responses than are ANSD subjects, again not supporting the hypothesis that a loss of neural responses could be responsible for the large SP in MD subjects.

Because the data in Figures 5A,B are not separated by the size of the SP, it is still possible that the cases with the large negative SPs were deficient in neural activity compared to cases with small SPs. To test this, data from one frequency (750 Hz) where the percent ANN is plotted as a function of the SP is shown in Figure 5C. This frequency was chosen because of the number of cases with a high proportion of ANN (Figure 5A) and large negative SPs (Figure 3B). Similarly, in Figure 5D, the percentage of cases that had a CAP to a high frequency (2 kHz) are plotted for cases with large and small SPs. In both data sets, there was no trend for the evoked neural activity to decrease in cases with large SPs.

### Examples of multiple contributions to the summating potential in human subjects

**FIGURE 6:**
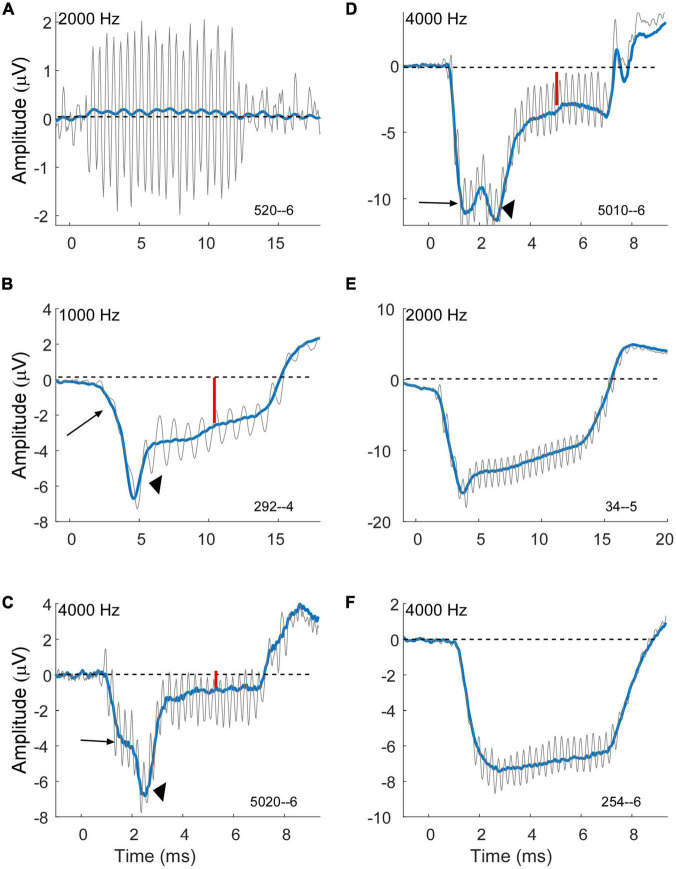
Variability of compound action potential (CAP) and summating potential (SP) morphology in electrocochleography (ECochG) waveforms of subjects with Meniere’s disease (MD). Gray is the response to condensation phase and blue is the sum of the alternated phases. **(A)** Example of a case with a strong cochlear microphonic (CM) but essentially no SP, indicative of little or no asymmetry in the hair cell output and little or no neural response. **(B–D)** Example of cases showing an early SP (long arrows) due to outer hair cells, a CAP (arrowheads) indicating neural involvement, and a steady state SP (small red arrows) that are presumably the sum of outer hair cell (OHC), inner hair cell (IHC) and neural contributions. Each example shows a different morphology of the CAP. **(E)** Example of a case with an early SP from OHCs, no obvious CAP, and then a steady state SP that is presumably the combination of OHC and later-arriving IHC contributions. **(F)** Example of a case with an early SP from OHCs and little change thereafter, suggesting limited IHC and neural contributions. All cases were at 90 dB nHL.

A large negative SP is a prominent feature in some cases for both MD and ANSD subjects who are receiving CIs, with MD being larger, but the distributions (Figure 3B) show the majority in both groups have an SP of less than about 1 μV. In some cases (e.g., Figure 6A), there does not appear to be an SP at all, rather an absence of one, which would be consistent with an absence of asymmetry from either hair cells or the auditory nerve in the response. In others (e.g., Figure 6B) a small SP is a driven response where a value near zero is the balance between contributions from different sources. We have attempted to derive metrics to separate an absent SP, indicative of no sources producing asymmetry in the AC response, but so far have been unsuccessful.

The cases in Figures 6B–F are examples of how contributions from multiple sources sum to produce the SP measured at steady state to tones. The large arrows in each case point to an “early SP” which is often more negative than the steady state (smaller red arrows). This early SP occurs before the CAP (arrowheads in B–D), and thus represents a negative polarity contribution with this recording configuration that is presumed to be from outer hair cells. It corresponds to the SP reported from clicks. The CAP has different latencies contributing to different profiles of response. Following the CAP, the steady-state SP becomes more positive, consistent with inputs from the inner hair cells and auditory nerve. In Figure 6E a CAP is not obviously present, but the profile produced by the negative early SP and more positive steady state creates a profile that can be difficult to distinguish from a CAP. In Figure 6F there is little indication of other than outer hair cells contributing since the early SP and steady state are the same. Different cases blend between each of these examples, so it is not easy to draw divisions between them for categorization.

## Discussion

We investigated whether neural dysfunction contributes to the large negative SP evoked by high intensity tone bursts in humans diagnosed with MD. Historically, especially in the clinical literature, the SP has been treated as a hair cell potential, with contributions from both inner and outer hair cells. Neural contributions to the SP have recently been shown in gerbils to provide a positive polarity contribution, such that its removal could in theory provide an alternative basis for the large negative SP in MD subjects (Pappa et al., 2019; Lutz et al., 2022). In addition, loss of neural potentials were associated with surgically induced endolymphatic hydrops in guinea pigs (Lee et al., 2020). However, the results of the present study were consistent with an increased asymmetry in the operating point rather than with neural dysfunction. While this is the main result, we will also consider some additional results observed about the SP from human CI subjects.

### Operating point asymmetry vs. neural dysfunction

To address whether neural dysfunction was contributing to the large negative SP in MD subjects, we used a control group of ANSD subjects known to have neural dysfunction. Our previous study on these ANSD subjects reported the enhanced negative SP primarily to high frequencies compared to control subjects that had other etiologies of hearing loss, and that correlated with a lack of a CAP in the ANSD subjects (Riggs et al., 2017). Through the comparison of the MD with these ANSD subjects we found three characteristics that indicate the mechanism for the enhanced SP in Meniere’s is not related to neural dysfunction.

First, the SP was disproportionally larger in the MD group, particularly to low frequencies, than in the ANSD subjects (Figure 3B). This was despite the AC responses of the ANSD group being large overall, frequently larger than those of MD group (Figure 3A).

Second, the magnitude of the AC response in MD, but not ANSD, subjects had a strong association with the size of the SP (Figure 4A). The difference between the two would not be expected if an increased negative SP was related to loss of neural function, as with ANSD subjects. A similar relationship was previously described for 1 kHz tone bursts where the size of the SP increased with the size of the CAP across MD subjects (Conlon and Gibson, 2000). The increasing SP with AC response magnitude is consistent with the overall increase in asymmetry of the operating point of the input-output functions of the transducer channels in the stereocilia of hair cells, i.e., the standard explanation for the increased SP in MD subjects. That is, with additional channels closed at rest, there will be a lowered SP threshold as saturation to one direction of motion begins earlier, and the SP will increase over a wider dynamic range until saturation in the other direction is reached. In contrast, a more symmetrical input output function will have an increased threshold and narrower dynamic range. In addition to the extra increment of SP from each cell with MD compared to ANSD, for a given spread of excitation as intensity is raised a larger number of cells will cross the threshold for asymmetric saturation. In contrast, in the ANSD subjects many or most of the additional hair cells added due to spread of excitation will be in their linear range of response and provide no increase in the SP with larger AC responses (Figure 4B). Finally, the increase in the negative SP from OHCs is not “balanced out” by an increase in positive SP from IHCs because the IHCs begin in a highly asymmetric state (80–90% channels closed at rest).

Finally, the neural responses in MD subjects were stronger to both low frequencies in the form of the ANN and high frequencies in the form of a CAP than in ANSD subjects (Figure 5). Obviously, this result is not consistent with a neural explanation for a large, negative SP in MD subjects. Thus, all factors considered in this study point to an increased operating point asymmetry due to EH as the mechanism for the large SP in MD subjects.

### Complexity of the summating potential

A large negative SP is a prominent feature in some cases for both MD and ANSD subjects who are receiving CIs (Figure 3B), with the SP in MD subjects being significantly more negative (LMM, *p* < 0.001). However, the distributions in both groups show most SPs to be small (<5 μV). A complexity is that in some cases an SP near zero indicates an absence of any asymmetry in the response, while in others it is clearly a driven response where the small value is how the contributions with different polarities from different sources balanced out. Unfortunately, we have not succeeded in developing metrics to distinguish these two distinct types of responses. In any event, the predominance of cases with small SPs in the groups studied here and in other groups of CI subjects (Riggs et al., 2017; Pappa et al., 2019) has implications for attempts to use the SP as a marker for the CF regions of contacts on an electrode (Helmstaedter et al., 2017). In principle, the polarity of the SP should flip as a CF region is traversed and the electrode moves from one side of a dipole to the other. This could work for the cases where the SP is prominent, but these are a minority. However, it may be that intracochlear recordings where the responses can be much larger than from the round window will show more cases to have an SP that is large enough to reliably demonstrate such changes.

In other examples the time course of the SP revealed which of multiple sources with different polarities, including IHCs, OHCs and the auditory nerve. These determinations were made in reference to recent results in gerbils using neurotoxins and ototoxins which showed different polarities and time courses for each of the sources (Pappa et al., 2019; Lutz et al., 2022). Given the differences in cochlear size, it is somewhat surprising that the results in human CI subjects so closely parallel those in gerbils. Presumably, this congruence represents the common biophysics of asymmetries produced by each source. However, there are two problems with this assumption. First, the reason for the different polarities of the SP from OHCs and IHCs at the round window are not clear. Pappa et al. (2019) suggested two possibilities, either the OHCs and IHCs had asymmetric operating points in different directions, e.g., more channels open than closed in OHCs and the opposite in IHCs, or there is a difference in source location, with the centroid of IHC contributions being more basally located than OHCs due to a greater asymmetry in the operating point. To the degree there is a geometric contribution to the different polarities it is possible but not necessarily expected to be similar in gerbils and humans. The second problem with an explanation of common biophysics between gerbils and humans is the case of the chinchilla, where the polarities of the SP from both OHCs and IHCs are positive (Zheng et al., 1997; Durrant et al., 1998). Chinchillas are also unique among studied species in that the IHCs are selectively affected by carboplatin, a common ototoxin used in many cancer treatments. Despite these important issues, the time courses of apparent contributions to the SP with different polarities are clear enough to show that, like the gerbil, there are interactions between the different sources that produce the steady-state SP to tones. It is interesting that in the hypothesized setting of EH in MD subjects the increased asymmetry of OHCs seems to dominate over effects of the contributions from IHCs or the auditory nerve.

### Limitations of the study

Patients within the MD group met the criteria for definite MD based on clinical diagnosis. However, this does not mean that they all had EH at the time of testing. Rather, this is inferred based on histologic studies where EH has been shown to frequently be present in ears with MD (Hallpike and Cairns, 1938; Paparella, 1984; Rauch et al., 1989; Salvinelli et al., 1999). Subjects not exhibiting EH at the time of testing would be expected to have less hair bundle asymmetry and thus small SPs at the time of testing.

Since its initial recognition (Starr et al., 1996), the ANSD diagnosis has been expanded to include various disruptions in auditory function originating from loss of IHCs, auditory nerve, their synapses or more centrally located sites of lesion. Furthermore, their ECochG electrophysiological phenotype has been shown to be heterogeneous (McMahon et al., 2008; Santarelli et al., 2008; Riggs et al., 2017). Thus, ANSD represents an imperfect a model of neural dysfunction, although it is the best available human model of preserved cochlear function in setting of neural dysfunction to test our hypothesis. In addition, the age distribution of the two groups is almost entirely non-overlapping. In general, cochlear responses decline with age (Chatrian et al., 1985; Starr et al., 2001). We do not think the cross-age comparison greatly affects the interpretation of the SP differences shown in this study. For the MD cases, which were from older subjects, there was a strongly increased SP compared to the young children with ANSD, despite the AC response being smaller on average. The SP in the ANSD children is much larger than other children or other adults receiving CIs (Riggs et al., 2017) so the increase in the MD subjects is larger than would be expected as the consequence of aging.

## Conclusion

The mechanism for the source of the large, negative SP magnitudes in ears diagnosed with MD is likely attributable to increased asymmetry in the hair cell transduction process due to EH, rather than to neural dysfunction.

## Data availability statement

The raw data supporting the conclusions of this article will be made available by the authors, without undue reservation.

## Ethics statement

The studies involving human participants were reviewed and approved by Institutional Review Board at the Ohio State University, Institutional Review Board at the University of North Carolina at Chapel Hill. The patients/participants provided their written informed consent to participate in this study.

## Author contributions

WR designed the study, participated in data collection and patient testing, and drafted and approved the final version of this manuscript. OA, DF, and TF participated in data analysis, provided critical comments on subsequent drafts, and approved the final version of this manuscript. TF, AM, VV, and MH participated in data collection and patient testing, provided critical comments, and approved the final version of this manuscript. All authors contributed to the article and approved the submitted version.
